# Hip and fragility fracture prediction by 4-item clinical risk score and mobile heel BMD: a women cohort study

**DOI:** 10.1186/1471-2474-11-55

**Published:** 2010-03-24

**Authors:** Daniel Albertsson, Dan Mellström, Christer Petersson, Hans Thulesius, Robert Eggertsen

**Affiliations:** 1 Department of Medicine/Public Health and Community Medicine/Primary Health Care, Sahlgrenska Academy at Göteborg University, Arvid Wallgrens backe, Göteborg, Sweden; 2 R&D Center, Kronoberg County Council, Jakob Lunds väg 2, Växjö, Sweden; 3 Department of Geriatrics, Sahlgrenska universitetssjukhuset/Mölndal S3, Mölndal, Sweden; 4 Mölnlycke Primary Health Care and Research Center, Ekdalavägen 2, Mölnlycke, Sweden

## Abstract

**Background:**

One in four Swedish women suffers a hip fracture yielding high morbidity and mortality. We wanted to revalidate a 4-item clinical risk score and evaluate a portable heel bone mineral density (BMD) technique regarding hip and fragility fracture risk among elderly women.

**Methods:**

In a population-based prospective cohort study we used clinical risk factors from a baseline questionnaire and heel BMD to predict a two-year hip and fragility fracture outcome for women, in a fracture preventive program. Calcaneal heel BMD was measured by portable dual X-ray laser absorptiometry (DXL) and compared to hip BMD, measured with stationary dual X-ray absorptiometry (DXA) technique.

**Results:**

Seven women suffered hip fracture and 14 women fragility fracture/s (at hip, radius, humerus and pelvis) among 285 women; 60% having heel BMD ≤ -2.5 SD. The 4-item FRAMO (Fracture and Mortality) Index combined the clinical risk factors age ≥80 years, weight <60 kg, prior fragility fracture, and impaired rise-up ability. Women having 2-4 risk factors showed odds ratio (OR) for hip fracture of 5.9 and fragility fracture of 4.4. High risk group hip fracture risk was 2.8% annually compared to 0.5% for the low risk majority (69%). Heel BMD showed hip fracture OR of 3.1 and fragility fracture OR of 2.6 per SD decrease. For 30 DXA assessed participants mean hip BMD at -2.5 SD level corresponded to a lower BMD at the heel. Five of seven hip fractures occurred within a small risk group of 32 women, identified by high FRAMO Index + prior fragility fracture + heel T-score ≤-3.5 SD.

**Conclusions:**

In a follow-up study we identified high risk groups for hip and fragility fracture with our plain 4-item risk model. Increased fracture risk was also related to decreasing heel BMD in calcaneal bone, measured with a mobile DXL technique. A combination of high FRAMO Index, prior fragility fracture, and very low BMD restricted the high risk group to 11%, among whom most hip fractures occurred (71%). These practical screening methods could eventually reduce hip fracture incidence by concentrating preventive resources to high fracture risk women.

## Background

Hip fracture (HF) is a common and severe trauma, with a 23% life time incidence in Swedish women[1-3]. More than 90% of HF occur as a result of falls[4]. Prevention of falls and/or fractures for these women can be achieved by walking and mobility training, tobacco use avoidance, home hazard reduction[5-11], pharmacological treatment[12-21], and probably by use of hip protectors[22,23].

HF risk group identification has been more fracture predictive by combining several clinical risk factors than by bone mineral density (BMD) assessment alone[24-27]. However, additional BMD assessments further improved fracture prediction[24,28,29].

Osteoporosis is diagnosed[1] when finding low BMD compared to a reference population of young healthy women. BMD values below T-score -1.0 SD is defined as osteopenia and from T-score -2.5 SD and below as osteoporosis. Osteoporosis combined with previous fragility fracture is defined as established osteoporosis. A typical fragility fracture usually occurs at distal radius, proximal humerus, pubic and ischial pelvic bones, hip or vertebrae after a low-energy trauma. BMD assessment with stationary Dual X-ray absorptiometry (DXA), rarely available in Swedish Primary Health care (PMC), has been evaluated for both fracture prediction and pharmacological treatment effects[30]. Optimal HF risk prediction is achieved by the DXA, measured on the hip (at "femoral neck" or "total hip") with Relative Risk (RR) at 2.6 per age-adjusted SD decrease[27,31,32]. HF prediction was slightly lower (RR = 2.0) when calcaneal heel BMD was assessed with older X-ray techniques, although the difference to assessment at hip was non significant[31]. For vertebral fracture risk, spine BMD assessment has better prediction or was in line with hip assessment[27,31].

Osteoporosis prevalence varies for different populations or techniques, but it also depends on the number of measured sites. DXA assessed hip osteoporosis among Swedish women aged 70-84 was 28-47%[33]. Hip osteoporosis prevalence among white US women aged 70-79 and ≥80 years was 24.5% and 47.5% respectively[31,34], while choosing the lowest BMD of either hip, spine and mid radius the prevalence increased to 38.5% and 70.0%[34]. There is a need for improved fracture prediction in Primary Health Care (PHC), to identify individuals at high fracture risk and prevention needs, and to protect women at minimal fracture risk from unnecessary investigations and treatment[33].

A simple clinical 4-item risk score has shown 81% sensitivity for two-year HF risk[35,36]. Additional BMD assessment may narrow that risk group even more, increasing the gain of bone strengthening therapy. A portable and easily handled BMD measuring device, Dual X-ray absorptiometry and Laser (DXL) technique, could enable practical assessment in PHC[37]. Previously, DXL assessment has shown sensitivity/specificity of 80% and 82% versus osteoporosis identification by DXA assessment[37]. A retrospective DXL study showed increased fracture history among women having low BMD[37,38] but prospective fracture prediction from DXL measure has not yet been demonstrated.

Our research question was "Does 4-item clinical risk score or DXL assessed heel BMD, alone or in combination, predict HF or fragility fracture (FF)?" We therefore decided to evaluate the two-year HF and FF risk in an elderly female population, involved in a fracture preventive programme.

## Methods

### Study population

This population-based PHC study included 285 of 390 women (73%) who answered a questionnaire and performed BMD assessments in 2003, and were alive at follow-up in 2004.

All 285 women for two years were part of an intervention group in a controlled fracture preventive study[35,39]. Clinical 4-item risk models were previously validated for fracture prediction during 2002-2003, based on survey data from altogether 1248 women followed during two years[35].

### Questionnaire

In September 2003 while measuring BMD, 285 women in the intervention area answered 15 questions on fracture risk including age, weight, height, having fallen last year, ability to rise five times from a chair without using arms (recommended self-test), earlier fractures including results of vertebral X-ray, family history of fractures, smoking, cortisone medication, and living conditions (see Table 1).

**Table 1:** Questionnaire data and heel BMD measured on 285 women at 2003.

	285 women	
	
*Characteristics*	Replies of 285	Mean/N valid (%)*
***Continuous risk factors***		
		
*Heel BMD by DXL *^†^		
T-score - Low side (SD) ^†^	285	-2.7 ± 1.0
		
T-score - Left side (SD)	284	-2.5 ± 1.0
T-score - Right side (SD)	283	-2.6 ± 1.0
		
*Clinical risk factors*		
Age (years)	285	79 ± 5.3
Weight (kg)	284	68 ± 12
Height (cm)	276	160 ± 6.3
		
***Risk groups with binary risk factors***		
		
*Heel BMD by DXL *^†^		
T-score ≥ -1.0 (SD)	285	13 (5)
-1.0 > T-score > -2.5 (SD)	285	101 (36)
-2.5 ≥ T-score > -3.5 (SD)	285	103 (36)
T-score ≤ -3.5 (SD)	285	68 (24)
		
T-score ≤ -2.5 (SD) + prior fragility fracture ^‡^	283	70 (25)
T-score ≤ -3.5 (SD) + prior fragility fracture ^‡^	283	35 (12)
		
*Main clinical risk factors as combined*		
FRAMO Index ^§^	285	88 (31)
		
*Main clinical risk factors as single*		
Age ≥ 80 years	285	108 (38)
Weight < 60 kg	284	69 (24)
Prior fragility fracture since age 50 ^‡^	283	94 (33)
Impaired ability to rise five times from chair	278	52 (19)
		
*Other possible risk factors*		
Any fall last 12 months	284	66 (23)
Cortisone medication more than 3 months	274	40 (15)
Living in residential care (vs community)	285	27 (9)
History of maternal hip fracture	274	33 (12)
Current smoking	284	12 (4)

* Percentage estimated on the valid participators and aritmetic mean value ± SD presented.

† Calcaneal BMD value measured bilateral by DXL-tecnique. Calculations usually made on the lowest value for either left or right heel.

‡ Prior fragility fracture is defined as fracture at hip, forearm, humerus, and vertebrae after 50 years of age.

§ FRAMO Index: High risk group has 2 of 4 risk factors of; Age ≥ 80 years, weight < 60 kg, prior fragility fracture and impaired ability to rise. Missing values recoded to low fracture risk.

A 4-item risk model, Fracture and Mortality (FRAMO) Index, evaluated previously in 2002-2003 among these women[35] was now recalculated using data from the current survey. Participants were classified at high fracture risk, when fulfilling at least two of four binary risk factors; age ≥80 years, weight <60 kg, previous fragility fracture since age 50 years (located at distal radius, proximal humerus, hip, or vertebrae), and impaired ability to rise.

Another earlier 4-item score, Risk Model II, with three items being the same but "having fallen the last year" was used instead of impaired ability to rise, was from 2003 used to direct the intervention types within the study population[35,39]. The fracture risk for that model was evaluated after previous study period 2002-2003, showing risk ratios at lower levels than for the FRAMO Index[36].

### Heel BMD assessment with DXL

In September to October 2003 we performed bilateral calcaneal heel BMD assessment in 285 of the 390 women in the intervention population. The portable Dual X-ray absorptiometry and Laser (DXL) Calscan device (article number: PN 031000)[40] uses two X-ray energies in combination with laser to determine the different absorptions of bone mineral, lean soft tissues and adipose tissues[37], with a precision CV% (coefficient of variability) in vivo of 1.24%[41]. DXL has no known physical side effects, was easily managed by two assistant nurses and the first author being trained by the equipment manufacturer (Demetech). Software DXL Calscan Workstation version 1.2 was used to calculate heel T-scores from the reference data population Women-Europe-1001[38].

In our data analysis of bilateral heel assessment we applied the lowest T-score of either side, because of substantial side differences of T-score 0.5 SD or higher among the 12% of women having substantially lower right heel than left heel BMD. This regime lowered the mean T-score with 0.18 SD as compared to a left side assessment only (p < 0.001).

### Hip and spine BMD assessment with DXA

Thirty consecutively chosen women accepted additional DXA assessment. The DXA assessments were performed at Ljungby hospital department of internal medicine measuring both hips (total hip and femoral neck) and the lumbar spine (between L2-L4). A Lunar DPX-Alpha #8225 device was used, estimating T-scores from the USA Femur Reference population and USA AP Spine Reference population[42]. Additional 11 women were DXA measured due to their heel T-score of ≤-3.5 SD.

We estimated BMD means and the correlation of paired samples between the sites measured. In further analyses we chose hip BMD as the reference measure site because HF is the most serious common fracture type[2,3] and since BMD at hip is the most HF predictive[31]. Also, in our sample we found heel to hip correlations above r = 0.68.

### Interventions

All 285 participants received a brochure with fall and fracture preventive advice in 2002. The 22% (62/285) of women, previously high risk classified (Risk Model II at 2001)[35] got repeated house calls by a nurse in 2003. Nurses gave advice about life style, fall prevention including safer home environment, and hip protectors. A physical home training programme was introduced. Seven percent continued home training until 2004 and 13% participated in physical group training.

After the BMD assessment all participants received information about their BMD and fracture risk. They also got fracture preventive advice and 72% were contacted by a physician individually. Following a major intervention in 2004, 41% reported on-going treatment with calcium and vitamin D and 13% taking a bisphosphonate.

### Fracture and mortality registration 2004 to 2005

We defined incident fragility fractures (FF) as fractures occurring in the hip, distal radius, proximal humerus, pubic bone, ischial bone and vertebrae during 2004-2005. Vertebral fractures were classified as incident if the radiograph report confirmed vertebral compression in women who had local pain. We identified all incident FF using diagnostic registers from the departments of orthopaedics, and from radiological film reports. We included diagnostic codes from ICD-10 [International Classification of Diseases], S72.00-72.21, S52.50-60, S42.20-21, S32.50, S32.80, S22.00 and S32.00.

Mortality data were collected from the National Swedish Population Register.

### Drop-outs

The original population of 435 women in the intervention area decreased by 5.2% annually since 45 died before the actual evaluation period 2004-2005, including two women having done BMD assessment.

The 27% (105/390) non-participants in this study were on average four years older, less able to rise up from chair, and were more in residential care at 2001, compared to the 285 participants.

After heel BMD assessment with DXL 46% (30/65) of consecutively chosen women accepted and performed an additional DXA investigation at hip and spine. Among women with low heel BMD 61% (11/18) performed the offered additional DXA assessment.

### Ethics

The Regional Ethics Committee at Lund University approved the study (LU 406-00). Also, the local radiation protection committee at Växjö Central Hospital approved the DXL and DXA screening. Each participant received oral and written study information and approved study participation by returning the questionnaire and undergoing BMD assessment.

### Statistical methods

Binary data were analyzed with Fisher's exact test, continuous normally distributed data by Student's t-test and asymmetric data by Mann-Whitney's U-test. Continuous or binary risk factors with binary outcome were also analyzed in logistic regression models. Missing replies for risk factor in risk models were recoded to the value for low fracture risk, to keep high study participation and avoid overestimation of risk ratios. Differences were regarded as significant when two-sided p-values were < 0.05. Data were analyzed in SPSS version 13.

## Results

### Baseline characteristics

Participation rate was 73% (285/390) and the age span 72-98 years with nine percent living in residential care, see Table 1. Heel BMD was assessed bilaterally for 282 women (99%) and for three women unilaterally Mean response rate to the four clinical items in FRAMO Index was 99%.

Around one fourth of the participants reported falls during the last year and one third reported prior fragility fracture (Table 1). Around one third were high risk classified by the FRAMO Index and each one of these four risk factors was found in 19%-38% of the participants. The total annual mortality rate was 4.2% and 24 women (8.4%) died during 2004-2005.

**Figure 1:**
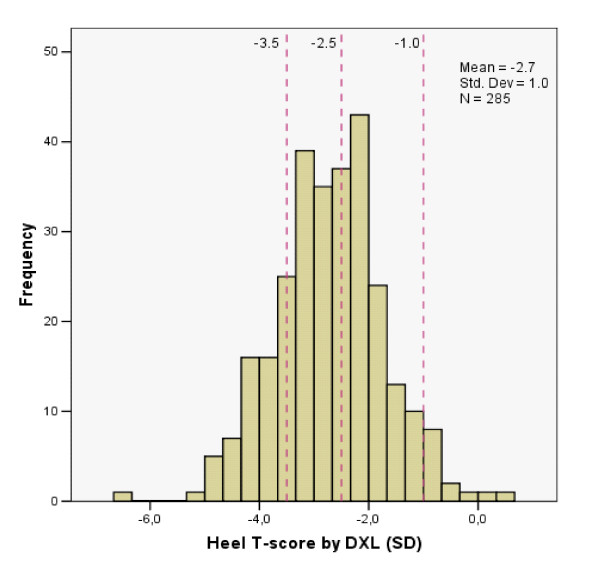
**Heel BMD by DXL technique on 285 women in PHC population**.

Only 5% had optimal heel BMD with T-score ≥-1.0, within the normal range of the younger reference population (Table 1 and Figure 1). Sixty percent had T-scores ≤-2.5 SD, 41% of these 172 women had previously suffered a fragility fracture.

### Risk factor evaluation for hip and fragility fracture

Seven women suffered HF and 14 women any FF (altogether 18 fractures; seven located at hip, four at distal radius, four at proximal humerus and three at pubic pelvic bone) during the study period, yielding an annual incidence of 1.2% and 2.5% respectively. Women who suffered HF and FF were 4-6 years older and had around 1.0 SD lower heel BMD, than those who did not fracture (p < 0.01 for all comparisons).

**Figure 2:**
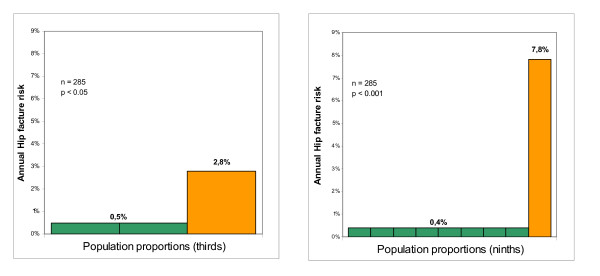
**Four risk factors (A) and four risk factors and heel BMD (B)**. **(A) **Hip fracture risk 2.8% annually for the 31% women having high FRAMO Index (orange column). Remaining 69% population had risk 0.4% (green column). **(B) **Hip fracture risk 7.8% annually for the 11% of population having high FRAMO Index + prior fragility fracture + BMD ≤-3.5 SD (orange column). Remaining 89% population had risk 0.4% (green column).

FRAMO Index showed HF and FF prediction with OR 5.9 and 4.4 respectively (see Table 2 and Figure 2A) and high risk classified women had an annual absolute risk for HF and FF at 2.8% and 5.1% respectively, yielding a HF sensitivity at 71% and specificity 70%. For the majority of women being at low fracture risk (69%) the corresponding HF and FF risk was 0.5% and 1.3% annually. Prior fragility fracture as a single item predicted HF risk and residential care was clearly related to increased HF and FF risk (see Table 2).

**Table 2:** Hip fractures and fragility fractures in 2004-2005 related to risk factors 2003 among 285 women.

	***285 subjects***						
	
		Hip Fractures (HF)			Fragility Fractures (FF)		
			
***Risk factors 2003***	N valid in risk group (% of n)	HF in risk group (n = 7)	OR HF (95%CI)*	p *	FF in risk group (n = 14)	OR FF (95%CI)*	p *
***Continuous risk factors***							
							
*Heel BMD by DXL *^†^							
T-score - Low side (SD) ^†^	285 (100)	7	3.1 (1.5-6.8)	0.004*	14	2.6 (1.5-4.6)	0.001*
							
*Clinical risk factors*							
Age (per year)	285 (100)	7	1.2 (1.04-1.32)	0.007*	14	1.1 (1.03-1.22)	0.01*
Weight (per kg)	284 (100)	7	1.0 (0.92-1.05)	0.6	14	1.0 (0.94-1.03)	0.4
Height (per cm)	276 (97)	7	0.9 (0.82-1.03)	0.14	14	0.9 (0.84-0.99)	0.03*
							
***Risk groups with binary items***							
							
*Heel BMD by DXL *^†^							
T-score < -1.0 SD	272 (95)	7	NA ^¶^	1.0	14	NA ^¶^	1.0
T-score ≤ -2.5 SD	171 (60)	6	4.1 (0.5-35)	0.2	12	4.2 (0.9-19)	0.05
T-score ≤ -3.5 SD	68 (24)	5	8.5 (1.6-45)	0.01*	8	4.7 (1.6-14)	0.006*
							
T-score ≤ -2.5 SD + FRAMO Index	74 (26)	5	7.6 (1.4-40)	0.01*	8	4.1 (1.49-12)	0.01*
T-score ≤ -2.5 SD + prior fragility fracture ^‡^	70 (25)	5	8.2 (1.6-43)	0.01*	7	3.3 (1.1-9.8)	0.05*
T-score ≤ -3.5 SD + FRAMO Index	42 (15)	5	16.3 (3.1-87)	0.001*	7	6.7 (2.2-20)	0.001*
T-score ≤ -3.5 SD + prior fragility fracture ^‡^	35 (12)	5	20.7 (3.8-111)	<0.001*	6	6.3 (2.0-19)	0.003*
							
*Main clinical risk factors as combined*							
FRAMO Index ^§^	88 (31)	5	5.9 (1.1-31)	0.03*	9	4.4 (1.4-14)	0.01*
							
*Main clinical risk factors as single*							
Age ≥ 80 years	108 (38)	5	4.4 (0.8-22)	0.11	9	3.1 (1.0-9.6)	0.05*
Weight < 60 kg	69 (24)	4	4.4 (1.0-20)	0.06	6	2.5 (0.8-7.4)	0.11
Prior fragility fracture since age 50 ^‡^	94 (33)	5	5.2 (1.0-28)	0.04*	7	2.4 (0.8-7.5)	0.13
Impaired ability to rise five times from chair ^||^	52 (19)	1	1.1 (0.1-9.9)	1.0	3	1.5 (0.4-5.6)	0.5
							
*Other possible risk factors*							
Any fall last 12 months	66 (23)	1	0.5 (0.1-4.6)	0.6	5	1.9 (0.6-5.9)	0.3
Cortisone medication more than 3 months	40 (15)	3	4.7 (1.0-22)	0.07	4	2.5 (0.7-8.4)	0.13
Living in residential care (vs community)	27 (9)	3	7.9 (1.7-38)	0.02*	5	6.3 (1.9-20)	0.006*
History of maternal hip fracture	33 (12)	0	NA ^¶^	1.0	1	0.6 (0.1-4.3)	1.0
Current smoking	12 (4)	0	NA ^¶^	1.0	0	NA ^¶^	1.0

¶ Not analysed [NA] Odds ratio when any cellvalue at 0.

† Calcaneal BMD value measured bilaterally by DXL-tecnique. Calculations usually made on the lowest value for left and right heel.

‡ Prior fragility fracture is defined as fracture at hip, forearm, humerus, and vertebrae after 50 years of age.

§ FRAMO Index: High risk group had 2-4 risk factors of Age ≥ 80 years, weight <60 kg, prior fragility fracture and impaired ability to rise from sitting position.

Missing values for these risk factors are recoded to low fracture risk.

|| The 2 of 7 women that sustained HF and did not reply their ability to rise, were recoded to low fracture risk, as able to rise.

* p < 0.05 significant difference. Binary item test by Fisher's Exact 2-tail. Continous item tested by logistic regression.

Lowered BMD increased the HF and FF risk by OR 3.1 and 2.6 respectively for each SD T-score decrease, as shown in Table 2. The age-adjusted BMD showed HF and FF risk by OR 2.3 (95%CI: 1.0-5.3, p = 0.05), and 2.2 (95%CI: 1.2-4.2, p = 0.01) respectively, in multiple logistic regression analyse. Higher age alone increased the HF and FF risk by OR 2.2 and 1.8 respectively, for every 5 years (Table 2).

For women having T-scores ≤-2.5 SD, HF and FF risk was significantly increased only when low BMD was combined with high FRAMO Index or a history of fragility fracture (Table 2). Isolated low BMD showed increased HF and FF risk at a T-score level ≤-3.5 SD, with OR 8.5 and 4.7 respectively, yielding a HF sensitivity at 71% and specificity 77%.

We identified a small high risk group by combining high FRAMO Index, prior fragility fracture, and T-score ≤-3.5 SD, finding 32 women suffering altogether five HF with an annual absolute risk of 7.8% and OR 23 (95%CI: 4.3-126), see Table 3. Among the remaining 253 women of low risk two HF occured, corresponding to a minimal HF risk at 0.4%, see Figure 2B. This risk factor combination shows a HF sensitivity at 71% and specificity 90%.

**Table 3:** Individual absolute risk of HF and FF annually, based on FRAMO Index, prior fragility fracture and heel BMD (T-score), alone or in combination (n = 285).

		HF 2004-2005			FF 2004-2005		
			
***Risk factor combinations***	Women at high risk (% of 285)	High risk (%)	Low risk (%)	p *	High risk (%)	Low risk (%)	p *
FRAMO Index	88 (31)	2.8	0.5	0.03 *	5.1	1.3	0.01 *
Heel BMD ≤ -2.5 SD	171 (60)	1.8	0.4	0.2	3.5	0.9	0.05
Heel BMD ≤ -3.5 SD + prior fragility fracture + FRAMO Index	32 (11)	7.8	0.4	<0.001*	9.4	1.6	0.002*

* Exact P-value as Fisher's two-sided test for binary variables, p < 0.05 as a significant difference.

### DXL level related to DXA assessment

The 30 consequently women whose BMD was measured with both DXL and DXA technique had a mean T-score at heel of -2.7 SD, being lower compared to means for hip (total hip -1.4 SD and femoral neck -2.0 SD) and to lumbar spine (-1.3 SD, p < 0.001 for all comparisons as paired differences). The BMD correlation was significant between heel and hip (total hip 0.71 and femoral neck 0.68, with p < 0.001) but for the lumbar spine correlation was low and non-significant (0.26, with p = 0.17).

**Figure 3:**
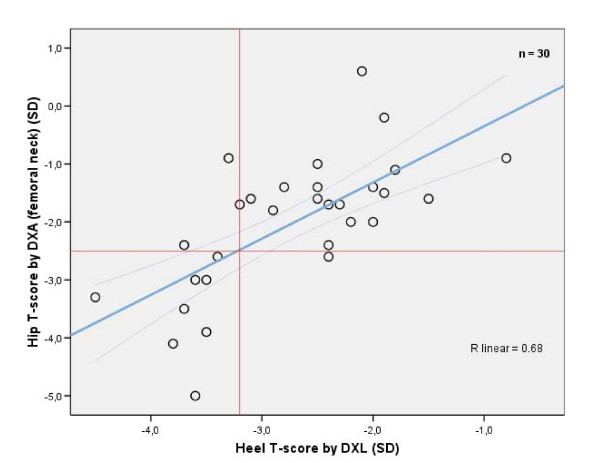
**T-score correlation for heel BMD compared to hip on 30 women**. The linear regression line crosses hip osteoporosis level -2.5 SD at the mean heel T-score of -3.2 SD, with the 95%CI of means -2.9 to -3.6 SD. Only one woman with high heel T-score >-3.2 SD had hip osteoporosis. Three women with low heel T-score ≤-3.2 SD did not have hip osteoporosis.

Scatter plot in Figure 3 illustrates BMD at heel compared to hip, shows that hip osteoporosis level (-2.5 SD at femoral neck) corresponded to a mean heel T-score of -3.2, with 95%CI: -2.9 - -3.6 SD at that DXA level. Applying that heel T-score threshold at -3.2 SD meant a hip osteoporosis sensitivity of 89% and specificity of 86%. Ninety percent of these women (27/30) were within the 95%CI limits of agreement[43].

Important risk factors for low BMD was equally distributed between these 30 DXA investigated women and the remaining 255 DXL investigated participants, showing DXL T-score at -2.72 vs -2.71 SD (p = 0.95), mean age 80.1 vs 78.9 years (p = 0.32), mean weight 68.6 vs 68.4 kg (p = 0.94), and 33% with a history of fragility fracture in both sub populations.

## Discussion

### Main findings

In this population-based study of 285 elderly women seven HF and 14 FF occurred during a two-year period. The practical 4-item FRAMO Index again confirmed fracture prediction with a six-fold increased risk for HF[35]. The high-risk classified women had an absolute HF risk of 2.8% annually, but for the majority of women (69%) having low risk it was only 0.5% (Figure 2A). As expected, fractures increased at higher age with doubled HF and FF risk for every five years age increment[44].

We found a very high absolute HF risk among the 11% (32/285) women at very low heel T-score ≤-3.5 SD in combination with high FRAMO Index and prior fragility fracture (Figure 2B). The remaining 89% of the population had low HF risk.

Only 5% of this elderly population had BMD above -1.0 SD assessed by heel DXL. The majority (60%) had T-score ≤-2.5 SD, despite age around 79 and being healthier than dropouts.

Prospectively evaluated population-based HF and FF risk was more than doubled for every SD decrease. For women having T-scores below the -2.5 SD threshold, the fracture risk was confirmed only when they had additional clinical risk factors (Table 2)[24]. This emphasises the importance of evaluating clinical risk factors at BMD assessment, before starting pharmacological treatment[25,29,45].

Hip osteoporosis level corresponded, in a representative DXA assessed subgroup, to a lower heel BMD around -3.2 SD, indicating a threshold within the 95%CI -2.9 to -3.6 SD (Figure 3).

### Other studies

Our now revalidated clinical 4-item FRAMO Index 2004-2005 showed a HF risk of 5.9 and a FF risk of 4.4. These risks are similar to those of the previous study period 2002-2003 (HF and FF risk at 7.5 and 6.7 respectively)[35]. The positive predictive two-year FRAMO Index value for HF was now 5.1%, equivalent with our previous study (5.4%)[35] and another extended 6-item model (5.6%)[29].

The DXL measured prevalence of "heel osteoporosis" (≤-2.5) was as high as 60% in this Swedish women population, average age 79 years. Hip osteoporosis prevalence in white US women at similar ages was 24.5-47.5% measured with the DXA reference method[34].

Among the 30 consecutive chosen women in our study being DXA assessed, we found osteoporosis among 30% (9 of 30, see Figure 3), finding the same prevalence as in a previous DXA assessment study[34], see further comments below[46].

Age-adjusted HF and FF risk showed an OR of 2.2-2.3 per SD decrease, with our mobile DXL technique, equal in HF prediction as compared to older heel BMD assessment X-ray techniques (RR = 2.0)[31]. With stationary hip assessed DXA the HF prediction showed RR at 2.6 (relative risk) per SD decrease[31], a method being evaluated for both fracture prediction and pharmacological prevention. In our study the extended bone strengthening therapy at low BMD possibly prevented some fractures, which would lower actual fracture ratios.

Mean heel DXL T-scores were significantly lower compared to DXA (-1.3 vs total hip and -0.7 to femoral neck) for a representative subgroup in our study. These results were close to findings in another study (-1.1 and -0.5 SD respectively)[46], to take into account when defining osteoporosis using DXL assessed BMD at the heel. Another study suggested the use of specific DXL T-score threshold intervals, either for treatment or for further DXA assessment[47].

Still, fracture preventive treatment should be based on the absolute fracture risk, mainly dependent on clinical risk factors, such as age, gender, prior fragility fracture, heredity, cortisone medication, etc[20,24-27,33]. For clinical risk groups BMD assessment is valuable[19,20,24,28,29] before specific bone enhancing therapy.

### Limitations

Our study was small with few fractures occurring during the two-year observation period. This gives a wide 95%CI for the binary variables especially, contributing to large OR variations for the single risk items as well. On the other hand, items causing significant results usually showed high OR, identifying potential strong risk factors. We combined several risk factors into our risk model, which stabilizes and reduces the influence of random variation. Despite few fracture outcomes our main findings on risk estimates were close to other study results, evaluating HF prediction with FRAMO Index and heel BMD with DXL method [31,36]. The combination of these four clinical risk factors and very low heel BMD showed very high OR and was strongly significant (p < 0.001), 95%CI being wide but the lower limit still above four. Enhanced HF prediction by applying BMD on clinical risk groups is found in other studies[24,28], although the more precise OR of our risk factor combination with BMD has not been determined before.

The population in this study was involved in a fracture prevention programme in 2003. Lower fall tendency and improved up-rise ability were reported during 2004[39] which to some extent lowered these risk model items and maybe also the fracture prediction of the risk model itself.

Two of the seven women who acquired HF during the observation period did not report their ability to rise in the 2003 survey, while in 2001 they reported impaired ability to rise. Had they reported the same ability in 2003 as in 2001, this would have adjusted the risk estimate for HF upwards.

### Further studies

Fracture prediction using the FRAMO Index alone and in combination with portable BMD DXL assessment ought to be repeated in larger urban populations[36], also of non-Scandinavian origin both for men and women to delimit the optimal thresholds for fracture prediction.

### Implications

In this pilot study we reassessed the practical clinical FRAMO Index and confirmed its fracture predictive ability[35]. This supports the use of clinical risk factors as a simple screening tool in a PHC population[36]. Moreover, it identifies the majority of elderly women at low HF risk, thus with minor need for specific fracture prevention[35].

A high FRAMO Index together with a low heel BMD (T-score ≤-3.5 SD), and a previous fragility fracture identified a subgroup of women with a very high risk of fracture. This selection optimized fracture prediction. Clinical risk factors and mobile heel BMD assessment combined seems to delimit women at marked fracture risk, and identifies a large majority of women being at minimal risk, especially for HF (Table 3). This seems feasible since a portable DXL instrument was practical to use for bilateral heel BMD screening in PHC. If these results are confirmed in larger studies this screening procedure could concentrate HF prevention to persons at very high risk. This would lower prevention treatment costs and side-effects of unnecessary prevention.

## Conclusions

In this population-based pilot study of 285 elderly women we re-identified high risk groups for hip fracture (HF) and fragility fracture (FF) by prospectively using the practical clinical 4-item FRAMO Index, with results similar to our previous study. We found that the HF and FF risk increased when heel BMD in calcaneal bone was low. We used the mobile heel DXL technique that predicted HF with the same accuracy as older heel BMD X-ray techniques. We thus identified a small group of women (11%) that sustained most HF (5 of 7) by using a combination of a high FRAMO Index, prior fragility fracture, and heel determined BMD below -3.5 SD. Our plain clinical 4-item screening method for hip fracture could improve HF prevention, by directing more resources to the women at actual high risk. Combining the 4-item screening method with heel BMD assessment seemed to improve the fracture prediction, although this needs to be tried in larger urban populations.

## Abbreviations

BMD: bone mineral density; CI: confidence interval; DXA: dual X-ray absorptiometry; DXL: Dual X-ray absorptiometry and Laser; FF: fragility fracture; FRAMO Index: Fracture and Mortality Index or Risk Model I; HF: hip fracture; OR: Odds Ratio; PHC: Primary Health Care; RR: Relative Risk; SD: standard deviation; T-score: Defined as the discrepancy in SDs related to mean BMD among a young healthy women reference population; vs: versus.

## Competing interests

DA was employed as general practitioner in the study area during the study period and gave medical treatment to some of the participant. The remaining authors declare that they have no competing interests.

## Authors' contributions

DA did conduct the study and was main contributor of design. He did the questionnaire construction, data collection, performed data analysis and drafted the manuscript. DM and CP contributed substantially to study design and manuscript. HT revised the manuscript critically for important intellectual content. RE was involved in study design during the whole study period. He also took part in interpretation of data and drafting of the manuscript. All authors read and approved the final manuscript.

## Pre-publication history

The pre-publication history for this paper can be accessed here:

http://www.biomedcentral.com/1471-2474/11/55/prepub
